# A Novel Nomogram for Individualized Gonadotropin Starting Dose in GnRH Antagonist Protocol

**DOI:** 10.3389/fendo.2021.688654

**Published:** 2021-09-14

**Authors:** Yubin Li, Yuwei Duan, Xi Yuan, Bing Cai, Yanwen Xu, Yuan Yuan

**Affiliations:** ^1^Reproductive Medicine Center, The First Affiliated Hospital, Sun Yat-sen University, Guangzhou, China; ^2^Guangdong Provincial Key Laboratory of Reproductive Medicine, Guangzhou, China; ^3^Department of Obstetrics & Gynecology, National University Hospital, Singapore, Singapore

**Keywords:** IVF, ICSI, GnRH antagonist protocol, gonadotropin starting dose, ovarian sensitivity, nomogram

## Abstract

Controlled ovarian stimulation (COS) is one of the most vital parts of *in vitro* fertilization-embryo transfer (IVF-ET). At present, no matter what kinds of COS protocols are used, clinicians have to face the challenge of selection of gonadotropin starting dose. Although several nomograms have been developed to calculate the appropriate gonadotropin starting dose in gonadotropin releasing hormone (GnRH) agonist protocol, no nomogram was suitable for GnRH antagonist protocol. This study aimed to develop a predictive nomogram for individualized gonadotropin starting dose in GnRH antagonist protocol. Single-center prospective cohort study was conducted, with 198 women aged 20-45 years underwent IVF/intracytoplasmic sperm injection (ICSI)-ET cycles. Blood samples were collected on the second day of the menstrual cycle. All women received ovarian stimulation using GnRH antagonist protocol. Univariate and multivariate analysis were performed to identify predictive factors of ovarian sensitivity (OS). A nomogram for gonadotropin starting dose was developed based on the multivariate regression model. Validation was performed using concordance statistics and bootstrap resampling. A multivariate regression model based on serum anti-Müllerian hormone (AMH) level, antral follicle count (AFC), and body mass index (BMI) was developed and accounted for 59% of the variability of OS. An easy-to-use predictive nomogram for gonadotropin starting dose was established with excellent accuracy. The concordance index (C-index) of the nomogram was 0.833 (95% CI, 0.829-0.837). Internal validation using bootstrap resampling further showed the good performance of the nomogram. In conclusion, gonadotropin starting dose in antagonist protocol can be predicted precisely by a novel nomogram.

## Introduction

Women undergoing controlled ovarian stimulation (COS) are treated with gonadotropins. Ovarian reserve, follicle stimulating hormone (FSH) threshold, FSH metabolic clearance rate and ovarian sensitivity (OS) differ from patient to patient (1). Therefore, the gonadotropin starting dose selection and subsequent dose adjustment are often dependent on the clinical experience of specialists. Inappropriate doses of gonadotropin will result in ovarian hyperstimulation syndrome (OHSS) or iatrogenic poor ovarian response. Thus, it is important to provide a method for more objective selection of gonadotropin starting dose, in order to optimize the number of oocytes, and to achieve pregnancies more effectively and safely.

In 2003, Popovic-Todorovic et al. developed a nomogram for recombinant FSH (r-FSH) starting dosage based on age, smoking habits and ultrasound parameters including antral follicle count (AFC), ovarian volume and total Doppler score in GnRH agonist long protocol (2), which succeeded in increasing the proportion of appropriate ovarian responses and ongoing pregnancy rate compared with a standard dose of r-FSH of 150 IU/day (3). Later, an algorithm developed by Howles et al. on the basis of cycles from 1378 women, determined r-FSH starting dosage according to more commonly measured parameters including age, body mass index (BMI), basal FSH and AFC (4). Following studies suggested that the use of this algorithm achieved adequate oocytes, lower OHSS rate and comparable clinical pregnancy rate compared to the group of r-FSH 150 IU/day (5, 6). As the important role of anti-Müllerian hormone (AMH) in assisted reproductive technology (ART) was recognized over the past decade (7), La Marca et al. presented another nomogram based on age, AMH and basal FSH (8). A retrospective study conducted from the same group showed that this nomogram could obtain more appropriate starting dose of gonadotropin compared to that obtained according to clinicians’ experience (9). Progress has been made in the consistency between the nomogram and clinical practice. However, all these nomograms mentioned above were for GnRH agonist protocol.

GnRH antagonist protocol is one of the commonly used COS protocols, which has been widely applied in recent years. Since follicle unsynchronization is more prominent and the duration of stimulation is relatively shorter compared with the long GnRH agonist protocol, a proper starting dose may be more important in this protocol. To the best of our knowledge, no nomogram has been developed for calculating the starting dose of gonadotrophin in a GnRH antagonist protocol. With the update of ART and the emergence of artificial intelligence medicine, the establishment of a simplified programming relation between the comprehensive assessment of ovarian reserve and the starting dose of gonadotropin is of great clinical significance.

In the present study, we developed a nomogram to determine the gonadotrophin starting dose in a GnRH antagonist protocol.

## Materials and Methods

### Ethics Statement

This study was approved by the institutional review board on 17th of March 2015 (2015 –35) and was registered in the Chinese Clinical Trial Registry with the registration number ChiCTR1800015081.

### Study Design and Setting

This is a prospective cohort study of women undergoing COS with a GnRH antagonist protocol, who started their IVF/ICSI cycles at the Reproductive Medicine center of the First Affiliated Hospital of Sun Yat-sen University between April 2018 and July 2018.

### Participants

A total of 198 women were included in our study. The inclusion criteria were as follows: [1] women received first IVF/ICSI treatment with a GnRH antagonist protocol; [2] 20-45 years of age; [3] at least six months from the last birth, without lactation, if there was a history of pregnancy. Women receiving mild stimulation were excluded. Women with diseases of the endocrine system such as the pituitary, thyroid, adrenal, and pancreas disorders; polycystic ovary syndrome; endometriosis; surgical history of ovary and a history of pelvic radiotherapy and/or chemotherapy were excluded. Women who took oral contraception or other drugs that may affect hormone secretion within three months were also excluded.

### GnRH Antagonist Protocol

All women underwent COS with GnRH antagonist fixed protocols. On the second day of the menstrual cycle, bilateral antral follicles (<10mm) were counted using transvaginalsonography, and women started COS treatment with gonadotrophin (Gonal-F, Merck Serono Europe Ltd or Puregon, N. V. Organon). The principle of selecting starting dose of gonadotrophin in our center was 150 IU/day for a woman with age≤ 34-year-old, BMI< 24 kg/m^2^, 6≤ AFC<15, and dosage would be increased if the woman was older (age≥ 35-year-old) or heavier (BMI≥ 24 kg/m^2^) or with poorer ovarian reserve AFC<6 or basal FSH>10 IU/L or AMH< 1 ng/ml. On the contrary, the dosage would be reduced if the woman was thinner (BMI<19 kg/m^2^) or with better ovarian reserve (AFC≥ 15 or AMH≥ 4 ng/ml). Actual dosage adjustment depended on clinical experience of the treating physicians. GnRH antagonist (Cetrorelix, BaxterorGanirelix, N.V.Organon) was added on the 5^th^ or 6^th^ stimulation day. When there were at least three follicles with the diameter >17 mm or at least two follicles >18 mm, ovulation was induced with human chorionic gonadotropin (Chorionic Gonadotrophin for Injection, Livzon). Ovum retrieval was performed 34-36 hours after ovulation induction.

### Serum AMH, FSH, LH, Estradiol, P, and T Level

Blood samples were collected before the beginning of COS on the second day of the menstrual cycle to determine the serum AMH and basal sexual hormone (FSH, LH, E_2_, P and T) levels. Beckman Coulter Access Immunoassay System based on chemiluminescence was applied to detect these hormones, and was operated strictly according to the instruction. The quality control results for all hormone detection items were within the target range.

### Outcome Measures

The primary outcome was the OS [numbers of oocytes/starting gonadotropin dose (IU)].

### Statistical Analysis

Mean and standard deviation (SD) were used for normally distributed variables. Univariate analysis was performed to identify predictive factors of OS. Variables with *P* value less than 0.1 were further assessed by backwards stepwise multivariate linear regression, using Wald *P*<0.05 for entry and *P*>0.1 for removal. A final multivariate linear model for OS was developed. The Post-hoc power analyses were conducted using G*Power (version 3.1.9.2.).

A predictive nomogram for gonadotropin starting dose was developed based on the results of multivariate analysis and the formula for OS (OS=numbers of oocytes/starting gonadotropin dose). In four women, the number of oocytes obtained was zero, so the OS was zero, which could not be used to calculate the gonadotropin starting dose. Thus, data of 194 women were used to establish the model.

Validation of the nomogram was performed using concordance statistics, and was measured by concordance index (C-index). For sensible models, C-index varies between 0.5 and 1.0 (the higher the better) (10).

To reduce overfit bias which would overestimate the accuracy of the nomogram, internal validation was performed using bootstrap re-sampling. Bootstrapping repeated (500 times) the process of drawing samples with replacement from the original data set. The model was corrected in the repeating procedures of developing a model in bootstrap samples and testing the model in those subjects not included in the bootstrap sample. The original R^2^ and mean square error (MSE) of the original regression model were compared with the corrected R^2^ and MSE. The closer the original and corrected statistics, the better the fit of the regression model.

The statistical analysis mentioned above was performed using IBM SPSS Statistics for Windows (version 23.0) and R (version 3.3.4). The *P* value <0.05 was considered statistically significant.

## Results

### Baseline Characteristics

**Table 1** outlined the baseline characteristics of women. The mean maternal age was 32.54 ± 5.44. The median serum AMH was 2.94 (1.18, 5.72) ng/ml. The median number of AFC was 8.0 (3.0, 13.0).

**Table 1:** Basline characteristics.

Variables	n	
Age (years)	198	32.54 ± 5.44
BMI (kg/m^2^)	198	21.22 (19.50, 23.36)
AMH (ng/ml)	198	2.94 (1.18, 5.72)
Basal E_2_ (pg/ml)	198	37.00 (28.00, 50.25)
Basal FSH (IU/L)	198	5.42 (4.57, 7.22)
Basal LH (IU/L)	198	3.29 (2.31, 5.10)
Testerone (ng/ml)	197^#^	0.30 (0.24, 0.41)
AFC	198	8.0 (3.0, 13.0)

AFC, antral follicle count; AMH, anti-Müllerian hormone; BMI, body mass index; E_2_, estradiol; FSH, follicle stimulating hormone; LH, luteinizing hormone. ^#^Eliminating one outlier value.

### IVF/ICSI Outcomes

**Table 2** showed the outcomes of IVF/ICSI. The median gonadotrophin starting dose was 225 IU/day, ranging from 100 to 450 IU/day. The average number of retrieved oocytes was 13.18 ± 10.66. Thus, the average OS was 0.0768 ± 0.0792.

**Table 2:** Outcomes of IVF/ICSI cycles.

Variables	n	
Gn starting dose (IU)	198	225.00 (150.00, 300.00)
Duration of stimulation	198	9.00 (8.00, 10.00)
Total dose of gonadotropin (IU)	198	1800.00 (1200.00, 2475.00)
No. of retrieved oocytes	198	13.138 ± 10.66
Ovarian sensitivity	194	0.0768 ± 0.0792
No. of 2PN oocytes	194	7.0 (3.0, 12.0)
No. of day 3 utilizable embryos*	194	4.0 (2.0, 7.0)

*At day 3 after fertilization, the embryos were scored based on morphology. The utilizable embryos were defined as normal fertilized embryos with four blastomeres and fragmentation<10%, and normal fertilized embryos with five or more blastomeres and fragmentation<25% on day 3. Gn, gonadotropin.

### Ovarian Sensitivity and Ovarian Reserve Indexes

Univariate linear regression analysis was performed to identify ovarian reserve indexes that correlated with OS. Serum AMH level (*P*<0.001) and AFC (*P*<0.001) were considered as positive independent factors, while age (*P*<0.001), basal serum FSH level (*P*<0.001), and BMI (*P*=0.04) were recognized as negative independent factors. Multivariate analysis demonstrated that serum AMH level, AFC, and BMI were independent factors for OS (**Table 3**). The multivariate regression model accounted for 59% of the variability of OS (adjusted *R^2 =^* 0.59). The Achieved Power of the test was > 0.99.

**Table 3:** Predictors of OS in univariate and stepwise multivariate linear regression analysis.

Variable	Univariate			Multivariate			
	Regression coefficient	Standard error	*P*	Regression coefficient	Standard error	*P*	Adjusted *R^2^*
Age	-0.0063	0.0010	<0.001	–	–	–	
BMI	-0.0035	0.0017	0.041	-0.002823	0.001106	0.012	
AMH	0.0149	0.0010	<0.001	0.012997	0.001026	<0.001	0.59
bFSH	-0.0067	0.0014	<0.001	–	–	–	
AFC	0.0050	0.0006	<0.001	0.002407	0.000511	<0.001	

AFC, antral follicle count; AMH, anti-Müllerian hormone; BMI, body mass index; bFSH, basal follicle stimulating hormone.

### Predictive Nomogram for Gonadotrophin Starting Dose

**Figure 1:**
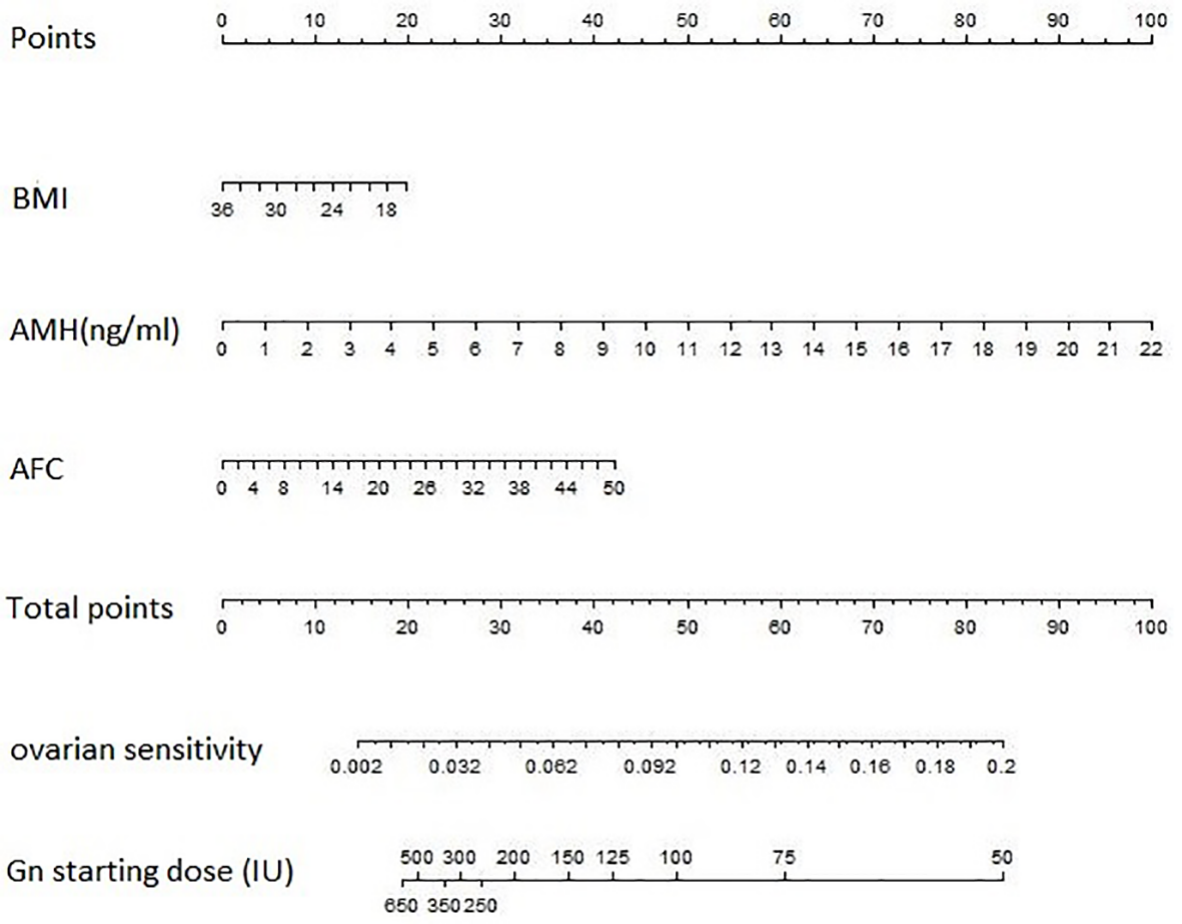
The predictive nomogram for gonadotrophin starting dose based on BMI, AMH and AFC. The nomogram is used by locating an individual patients’ value on each variable axis and then drawing a line upward to determine the number of points received for each variable value. Next, the sum of these numbers is located on the Total Points axis, and a line is drawn downward to the OS and gonadotropin starting dose axes to identify the predicted OS and the recommended Gn starting dose. AFC, antral follicle count; AMH, anti-Müllerian hormone; BMI, body mass index; Gn, gonadotropin.

On the basis that the optimal gonadotrophin starting dose was calculated by the optimal number of retrieved oocytes (we chose 10 as the optimal number)/OS, a nomogram (**Figure 1**) was developed to predict for gonadotrophin starting dose using the multivariate model for OS. The model for predicting OS was as follow:


$$0.061825-0.002823^{*}\text{BMI}+0.12997^{*}\text{AMH}+0.002407^{*}\text{AFC (BMI }[\text{kg}/{\text{m}}^{2}];\text{ serum AMH [ng}/\text{ml])}.$$

### Validation of Predictive Accuracy of the Nomogram

The C-index was 0.833 (95% CI, 0.829-0.837) in the concordance statistics of the multivariate regression model for OS, which represented excellent accuracy. During the bootstrap re-sampling, the model retained its excellent accuracy, returning a corrected R^2^ of 0.5696 and a corrected MSE of 0.0027, which was close to the original values (the original R^2^ = 0.5961, the original MSE = 0.0025).

## Discussion

COS is one of the most vital parts of IVF-ET. At present, no matter what kinds of COS protocols (GnRH agonist long protocol, GnRH antagonist protocol and GnRH agonist prolonged protocol, *etc.*) are used, clinicians have to face the challenge of selection of gonadotropin starting dose. Inadequate starting dose leads to insufficient follicular recruitment, which results in iatrogenic poor ovarian response. On the contrary, excessive starting dose is associated with excessive recruitment of follicles, leading to an increased incidence of OHSS. Furthermore, it may increase progesterone level during COS, and finally increases the cancellation rate of transfer in fresh cycles or decreases the pregnancy rate due to unsynchronized development of endometrium. In addition, high level of FSH was considered to be associated with chromosomal aberrations in oocytes (11). Therefore, choosing an appropriate starting dose has always been a challenge for clinicians. However, there were opinions that an individualized starting dose was not superior to a standard starting dose of 150 IU/day when focusing on the rate of live birth and cost-effectiveness (12–14). In fact, when paying attention to the rate of cycle cancellation, they found it higher in the standard dose group both within the overall strategy analysis (standard dose group *vs* individualized dose group: 14.5% *vs* 11.7%) and the RCT focusing on predicted poor responders (standard dose group *vs* individualized dose group: 20.3% *vs* 9.6%). It’s worth noting that the individual starting dose was chosen simply based on AFC in these studies. Whether women could benefit from a more individualized starting dose calculated by an effective algorithm is still unclear.

In this prospective cohort study, we developed a multivariate regression model including three independent factors (BMI, AMH, and AFC) for predicting individual OS. On the basis of this model, a predictive nomogram for optimal starting dose of gonadotropin in a GnRH antagonist protocol was further developed. The nomogram performed well in predicting starting dose, as the C-index was 0.833 (95%CI, 0.829-0.837). And this model was internally validated.

Women taking oral contraception within three months were excluded in our study as it may affect hormone secretion. Moreover, a systematic review from Cochrane library summarized that pretreatment with combined oral contraceptive pills lowered the rate of live birth or ongoing pregnancy rate in antagonist cycles (15). Previous ovarian surgery was one of the criteria for exclusion, for it. Operations may weaken the blood supply of ovaries, influencing the pharmacodynamics of Gn.

We chose 10 as the optimal number of retrieved oocytes. In the study of Verberg et al., it was found that the highest ongoing pregnancy rate per embryo transferred was associated with a median of 10 oocytes (16). The nomogram, published by La Marca et al. (8) was constructed after setting the optimal number of retrieved oocytes as nine, since nine was the average number of retrieved oocytes in that study. Sunkara et al. analyzed data from 400,135 IVF cycles, and demonstrated that the live birth rate was highest when the number of oocytes reached 15 (17). Magnusson et al. analyzed data of the recent decade (18). The study concluded that live birth rate after fresh cycles increased up to 11 oocytes retrieved while the cumulative live birth increased up to 20 oocytes retrieved, and the severe OHSS rate increased more rapidly when more than 18 oocytes were retrieved, and thromboembolic events occurs more frequently when more than 15 oocytes were retrieved. In addition, it is confirmed that supraphysiologic levels of estradiol and progesterone during COH could modify morphologic and biochemical features of endometrium, and consequently impair endometrial receptivity (19). In order to keep a balance between efficacy and safety, it is rational for us to set a goal of 10 oocytes.

Appropriate ovarian reserve markers are the basis of optimal ovarian response. The decrease in ovarian reserve is manifested in the reduction of the number of follicles that can be recruited, or accompanied by a decrease in the quality of oocytes. Age, AMH, AFC, and FSH are commonly used indicators to evaluate ovarian reserves in clinical practice. Recently, AMH has been recognized as one of the most sensitive indicators to evaluate the ovarian reserve (20, 21). Antral follicles are precursors of mature follicles, and the number of antral follicles can reflect the number of primordial follicles in the follicle pool. Clinicians may benefit from the AFC when estimating ovarian reserve before IVF (22). AMH and AFC are believed to be more effective in predicting ovarian reserve than basal FSH and other sex hormones. In addition, body weight is one of the factors that interfering gonadotropin secretion, and obesity is related to ovarian dysfunction (23). Serum level of gonadotropin entering the body is directly affected by the weight. Thus, the body weight is also recognized as an indicator predicting the optimal starting dose. Therefore, we chose age, BMI, serum AMH, basal FSH, and AFC as alternative predictive factors in this study.

The three independent factors (AMH, AFC and BMI) considered in our model are consistent with the most commonly used indicators for clinicians when selecting the starting dose. The CONSORT algorithm was established with factors of age, basal FSH, BMI, and AFC, without including the value of AMH. It is probably due to the fact that AMH was not widely applied for assessing ovarian reserve 10 years ago. AMH have been considered to be more accurate in predicting ovarian reserve than basal FSH in recent years. Therefore, the equation in the present study is an improvement over the algorithm developed 10 years ago.

The nomogram, published by La Marca et al., used age, AMH and basal FSH to predict optimal gonadotropin starting dose (8). It is debatable that this nomogram failed to introduce BMI, a recognized factor affecting dosage of drugs, as an independent variable. In addition, it was established based on the long GnRH agonist protocol, not the GnRH antagonist protocol in our study. Whether different kinds of protocols affect the introduction of variables deserves further analysis.

To the best of our knowledge, this is the first nomogram for starting dose of gonadotropin in a GnRH antagonist protocol. One of the advantages of the nomogram is that it is a weighted model, combining different predictive factors and predicting the starting dose more objectively. Furthermore, the factors included in the model are routinely measured in clinical practice, which makes the nomogram more easy-to-use.

This study has several limitations. Firstly, although the power of the test was good (> 0.99), the nomogram was developed based on the data obtained from a single center with a relatively low sample size, largely due to limited funding. Secondly, though validated internally, this model should be further validated externally with women evaluated at multiple centers. Moreover, although there was no restriction on BMI, women included in this study were tend to be thin with a small proportion of obesity (3 in 198 women with BMI>30kg/m^2^). Thus, the nomogram may be less effective when applying to obese population. Furthermore, FSH receptor polymorphism, which is a potential parameter for gonadotrophin selection (24), was not analyzed in this study. We are looking forward to taking the advantages of this factor for a more effective predictive model. Finally, the assays used to measure AMH may differ between laboratories. We used Beckman coulter Access AMH chemiluminescence detection kit to measure AMH in this study. However, apart from this assay, there are other forms of the assay, such as the original research assays, the DSL and Immunotechassays, the AMH enzyme-linked immunosorbentassay (ELISA) (25). Different assays have different specificity and sensitivity. This may affect the competence of widespread application of this nomogram.

## Conclusion

In conclusion, we developed a clinically practical nomogram for prediction of appropriate gonadotropin starting dose using a novel statistical method. Future work will focus on improvement of this model. Hopefully, it can be developed with artificial intelligence, and used widely and conveniently in clinical practice.

## Data Availability Statement

The raw data supporting the conclusions of this article will be made available by the authors, without undue reservation.

## Author Contributions

YL, YD, and XY contributed equally to study design, analysis, and manuscript drafting. BC contributed to execution and data analysis. YX and YY contributed to study design, revision, and final approval of the manuscript. All authors contributed to the article and approved the submitted version.

## Funding

The authors disclosed receipt of the following financial support for the research, authorship, and/or publication of this article. This work was supported by Grants from Guangzhou Science and Technology Project (201804020087), National Key Research and Development Program (2018YFC1003102), and National Science Foundation for Young Scientists of China (81100470).

## Conflict of Interest

The authors declare that the research was conducted in the absence of any commercial or financial relationships that could be construed as a potential conflict of interest.

## Publisher’s Note

All claims expressed in this article are solely those of the authors and do not necessarily represent those of their affiliated organizations, or those of the publisher, the editors and the reviewers. Any product that may be evaluated in this article, or claim that may be made by its manufacturer, is not guaranteed or endorsed by the publisher.
